# Recycled HDPE/Natural Fiber Composites Modified with Waste Tire Rubber: A Comparison between Injection and Compression Molding

**DOI:** 10.3390/polym14153197

**Published:** 2022-08-05

**Authors:** Ali Fazli, Tatjana Stevanovic, Denis Rodrigue

**Affiliations:** 1 Department of Chemical Engineering, Université Laval, Quebec, QC G1V 0A6, Canada; 2 Department of Wood and Forest Sciences, Université Laval, Quebec, QC G1V 0A6, Canada

**Keywords:** recycled HDPE, flax fibers, waste tire rubber, hybrid composites, coupling agent, molding conditions

## Abstract

With the objective of turning wastes into added-value materials, sustainable and fully recycled wood-plastic composites were reinforced by waste tire rubber particles to show balanced properties and potentially low-cost materials. Recycled high density polyethylene (rHDPE) was compounded (melt extrusion) with flax fiber (FF) and waste regenerated tire rubber (RR) to investigate the effect of mixing ratio, coupling agent (maleated polyethylene, MAPE) and molding process (injection and compression molding) on the properties of hybrid composites. In particular, a complete set of characterization was performed including thermal stability, phase morphology and mechanical properties in terms of tension, flexion and impact, as well as hardness and density. Adding 40 wt.% of flax fibers (FF) increased the tensile (17%) and flexural (15%) modulus of rHDPE, while the impact strength decreased by 58%. Substitution of FF by waste rubber particles improved by 75% the impact strength due to the elasticity and energy absorption of the rubber phase. The effects of impact modification were more pronounced for rHDPE/(FF/RR) compatibilized with MAPE (10 wt.%) due to highly improved interfacial adhesion and compatibility. The results also suggest that, for a fixed hybrid composition (FF/RR, 25/55 wt.%), the injection molded composites have a more homogenous morphology with a uniform distribution of well embedded reinforcements in the matrix. This better morphology produced higher tensile strain at break (12%) and impact strength (9%) compared to compression molded samples.

## 1. Introduction

There is still a growing interest for the production of biocomposites as natural fibers have emerged as low cost, highly available and environmental friendly reinforcement to produce sustainable composites for a wide range of applications, such as electroactive papers, membranes and barrier, floor protection and automotive parts [1,2]. Natural fibers, such as maple, hemp, flax, cotton and wood, have several advantages, including lightweight, high stiffness, biodegradability, recyclability and a wide range of types to replace carbon and glass fibers, as well as other synthetic fibers for reinforcement of polymer composites [3,4]. Due to biodegradablity and no negative effect on the environment, most of these plant fibers are classified as eco-friendly fibers [4]. However, the presence of high cellulose content with polar hydroxyl (–OH) groups makes it difficult to work with natural fibers due to poor compatibility between hydrophilic fibers and hydrophobic thermoplastic matrices. There are also other limitations, such as high moisture content (high water uptake in most composites), low fire resistance and durability, as well as low impact strength of the resulting natural fiber composites (NFC) [5,6].

Different studies have been conducted to improve the performance of NFC and determine the relation between composite properties with respect to fiber length, shape and, most importantly, interfacial adhesion with different matrices [7,8]. Natural fibers surface treatment using solution [5], silane coupling agents [7] and impregnation [8] can increase the interactions between the fillers and polymers to more efficiently transfer the applied loads and improve the NFC strength. Chimeni et al. [5] investigates hemp surface modifications using mercerization and maleated polyethylene (MAPE) treatment methods to improve interfacial bonding of hemp with linear medium density polyethylene (LMDPE). However, these modifications are expensive and not always eco-friendly due to the solvents used (mainly organics). This is why cost effective and environmentally friendly solutions to improve the compatibility between each phases in biocomposites must be investigated [1,9]. Once good adhesion is obtained, fiber addition results in higher tensile and flexural rigidity (modulus and strength), while a noticeable drop of elasticity and impact strength eventually leads to brittle NFC [7]. For example, Kazemi et al. [10] investigated the effect of wood sawdust addition to a recycled (post-consumer) polyethylene (PE)/polypropylene (PP) blend and observed substantial increase (15%) in the tensile modulus of the composite (265 to 305 MPa) upon addition of 20 wt.% natural fibers. In a similar work, Bar et al. [11] reported a 36% (8 to 11 GPa) and 40% (10 to 14 GPa) increase in tensile and flexural moduli by increasing the flax fiber (FF) content from 40 to 60 wt.% in PP, while the impact strength decreased by 20% (48 to 39 J/m^2^) due to non-homogeneous fiber distribution and poor interfacial adhesion. One way to solve the low impact resistance of NFC is to add an elastomer as an impact modifier. Several elastomers, such as ethylene-octene copolymer [12], natural rubber (NR) [13], ethylene propylene diene-monomer (EPDM) [14] or styrene butadiene rubber (SBR) [15], were used to increase the amount of energy absorbed before fracture through local deformation of the rubbery particles. Ruksakulpiwat et al. [13] reported a 66% (18 to 30 kJ/m^2^) and 133% (18 to 42 kJ/m^2^) impact strength increase of natural fiber (vetiver grass) reinforced PP composites by adding 30 wt.% of NR and EPDM, respectively. This improvement was attributed to the elastic behavior of the rubber phase with high toughness and elongation without permanent deformation.

Irrespective of its advantages, adding virgin rubber to NFC not only results in lower modulus, but also increases the cost of the resulting compounds [14]. For this reason, recycled rubber from waste tire crumbs can be used to manufacture fiber reinforced thermoplastic elastomers (TPE) with reduced raw material costs. Furthermore, from environmental point of view, growth of waste polymeric materials disposal (plastics and rubber) around the world indicates the need to develop proper methods to reuse waste materials and decrease the negative environmental effects [16]. For example, Kakroodi et al. [17] applied ground tire rubber (GTR) as an effective impact modifier to produce TPE composites reinforced with hemp. The results showed that adding 10 wt.% GTR into MAPE/hemp (50/50) composites increased by 50% (134.0 to 201.3 J/m) the impact strength of hybrid composites while tensile strength and modulus decreased by 32% (16.4 to 12.4 MPa) and 31% (292 to 222 MPa), respectively [17].

The low affinity between crosslinked GTR or partially regenerated tire rubber (RR) with most thermoplastic resins contribute to phase separation (incompatibility) and poor interface quality leading to inadequate stress transfer and failure at their interface [18,19]. According to previous works, the presence of a compatibilizer based on different copolymers, such as MAPE [14,20], styrene-ethylene-butylene-styrene (SEBS) [21], or styrene-ethylene-butylene-styrene-grafted maleic anhydride (SEBS-MA) [22], improved the interfacial adhesion to achieve better interactions between the fillers (fibers and/or rubbers) and the matrix resulting in higher mechanical properties.

Although formulation is an important parameter, the processing method is also important to control the final properties of complex polymeric systems. Extrusion, injection molding (IM), and compression molding (CM) or thermoforming (pressing) are the most used processes for manufacturing wood-plastic composites (WPC) [23]. Both CM and IM are frequently used as efficient and cost-effective molding processes for fabricating high-quality components. Although CM is easier to control/operate, it requires longer cycle times compared to IM [24,25]. For each method, a balance between processing conditions, such as pressure, temperature, molding time and heating/cooling rate, is essential to optimize the contact/interaction between the resin and the fillers with limited degradation (mechanical, thermal and oxidative) [26]. Since natural fibers, like most reinforcement, are prone to break-up (size reduction) during processing (due to fiber–polymer and fiber–fiber interactions), their dimensions (length, diameter, shape and aspect ratio) are modified resulting in different reinforcing efficiency [27]. Distribution/dispersion and orientation also depend on the processing methods and conditions [26].

Although a large body of literature is available on NFC and several studies were published on hybrid systems based on natural fillers and rubber particles (virgin or recycled) [5,22,28,29], there is a very limited number of studies on the use of recycled tire rubber (GTR/RR) to modify the properties of NFC [14,26], and even less information is available on the effect of the processing conditions and methods [24,26,27].

This study investigates the effect of both natural fiber (FF) and recycled rubber (RR) content on the thermal, morphological and mechanical properties of polymer composites prepared by different molding processes. As a result, fiber reinforced rubberized composites represent an opportunity for process of high volume of waste materials (plastic and tire) and production of more sustainable materials. An experimental optimization was performed to develop a specific phase morphology and achieve balanced properties and commercialization of sustainable composites. Here, MAPE is applied as a compatibilizer for both FF and RR to produce highly toughened hybrid biocomposites with high filler concentrations and balanced properties. Moreover, this study aims to evaluate the relation between the phase morphology and mechanical properties with respect to the molding process. In all cases, the compounds were prepared via melt blending (twin-extruder) and processed with via injection or compression molding for further analysis.

## 2. Materials and Methods

### 2.1. Materials

The matrix used for this study was post-consumer recycled high density polyeth-ylene (rHDPE) flakes from recycled solid HDPE bottles provided by Service de Consultation Sinclair (Drummondville, QC, Canada). This polymer has a peak melting temperature of 127.5 °C (ASTM D3418), a melt flow index (MFI) of 6.7 g/10 min (190 °C and 2.16 kg, ASTM D1238) and a density of 0.986 g/cm^3^ (ASTM D2856). The flax fibers (FF) were supplied by Biolin Research Inc. (Saskatoon, SK, Canada) and were sieved to keep only particles between 355 and 500 μm with density of 1.296 g/cm^3^. This average fiber size produced the best dispersion and mechanical performance compared with similar works [30]. The rubber particles (PI3.1.C), from regenerated car tire rubber (RR) particles, were kindly provided by Phoenix Innovation Technologies (Montreal, QC, Canada). RR particles, with an average particle size of 500 μm and a density of 1.184 g/cm^3^, were used as received. The coupling agent selected was MAPE, Epolene C-26 (Westlake Chemical Corp, Houston, TX, USA) with an average molecular weight of 65 kg/mol, a density of 0.920 g/cm^3^, an acid number of 8 mg KOH/g and an MFI of 8 g/10 min (190 °C and 2.16 kg).

### 2.2. Composite Production

Initially, the flax fibers (FF) were dried overnight in an oven at 90 °C. The rHDPE was compounded with FF (20 and 40 wt.%) and RR (20, 40, 60 and 80 wt.%) separately to produce hybrid biocomposites (rHDPE/FF) and TPE blends (rHDPE/RR). Although very high RR concentrations (up to 80%) were used, good homogeneity was achieved, but this limited the amount of FF that was possible to melt blended with rHDPE pellets (maximum concentration of 40%). Next, the fibers and rubbers were dry blended to reinforce the natural fiber TPE samples with different ratios as presented in Table 1. Furthermore, MAPE as a compatibilizer (10 wt.% based on the total filler weight fraction) was incorporated to the rHDPE/(FF/RR) blends including both reinforcements (FF and RR). For all cases, melt blending was performed using a co-rotating twin-screw extruder (Leistritz ZSE-27, Nürnberg, Germany) with a L/D ratio of 40 and 10 heating zones (die diameter of 2.7 mm). To minimize thermal degradation of the materials, the barrel temperature profile was fixed at 170 °C, while the screw speed was set at 100 rpm. The rHDPE and MAPE were introduced through the main feeder (zone 1), while the fillers (FF, RR or FF/RR) were added via a side-stuffer (zone 4) of the extruder (10 zone total) to prevent process overload and minimize thermo-mechanical degradation. To prevent high motor torque and die pressure associated with the high viscosity of RR containing blends, the total flow rate was fixed at 4 kg/h for all the blends. To cool down and solidify the extrudate, a water bath was used. The extruded samples were pelletized using a model 304 pelletizer (Conair, Cranberry Township, PA, USA) followed by drying (6 h in an oven at 80 °C) to eliminate any residual water for further processing. After drying, the final samples were produced by injection molding (IM) on a PN60 (Nissei, Japan) IM machine. The injection temperature profile was set as 175–170–170–160 °C (nozzle, front, middle and rear, respectively) with a mold temperature of 30 °C. The mold has four cavities to directly produce the geometries needed: two dumbbell shapes (type IV of ASTM D638) and two rectangular bars (width and thickness of 12.45 × 3.14 mm^2^ with two lengths of 80 and 125 mm). Different injection pressures (45 to 55 MPa) were applied depending on the blend composition. Samples were also prepared by compression molding (CM) on an automatic press (Carver, AutoFour/1512-PL,H, 3893, Wabash, IN, USA). Molding was performed at 170 °C by preheating the specimen for 3 min without pressure followed by 5 min under pressing (3 tons) followed by cooling under pressure to 60 °C using circulating water. The mold dimensions were 115 × 115 × 3 mm^3^ and the specimens were later cut into different geometries for mechanical characterization.

### 2.3. Characterization

#### 2.3.1. Thermogravimetric Analysis (TGA)

Thermal stability of the raw materials and the composites before molding were investigated via thermogravimetric analysis (TGA) on a Q5000 IR (TA Instruments, New Castle, DE, USA). A heating rate of 10 °C/min was applied between 50 and 700 °C. The tests were performed in air and nitrogen atmospheres to evaluate both thermal and oxidative resistance of the materials.

#### 2.3.2. Morphology

Scanning electron microscopy (SEM) was used to study the samples morphology and determine the interfacial contact between each phase. An Inspect F50 (FEI, Hillsboro, OR, USA) was used at 15 kV to take micrographs of the raw materials and cryogenically (liquid nitrogen) fractured surface of the compounds. The samples were previously coated with gold/palladium to be observed at different magnifications.

#### 2.3.3. Mechanical Testing

Tensile tests were conducted at room temperature according to ASTM D638 using a 500 N load cell and a rate of 10 mm/min on an Instron (Norwood, MA, USA) universal mechanical tester model 5565. At least five specimens produced by IM (type IV) and CM (type V) were used for each formulation. The average values of tensile strength (σY), Young’s modulus (E) and elongation at break (εb) are reported with standard deviations.

Flexural tests were performed on an Instron (Norwood, MA, USA) model 5565 with a 50 N load cell according to ASTM D790 at room temperature. Rectangular specimens produced via IM and CM with dimensions of 60 × 12.7 mm^2^ were tested with 5 repetitions in a three-point bending mode (span length of 60 mm) at a speed of 2 mm/min.

Notched Charpy impact strength was measured on a Tinius Olsen (Horsham, PA, USA) model 104 at room temperature according to ASTM D256. At least 10 specimens produced via IM and CM with dimensions of 60 × 12.7 mm^2^ were used for each compound. Before testing, all the samples were automatically V-notched on a Dynisco (Franklin, MA, USA) model ASN 120 m sample notcher 24 h before testing.

#### 2.3.4. Physical Properties

Hardness (Shore D) was determined by a model 307L durometer (PTC Instruments, Boston, MA, USA) with 10 measurements for each sample.

Density was determined by a gas (nitrogen) pycnometer Ultrapyc 1200e (Quantachrome Instruments, Boynton Beach, FL, USA). Each measure was repeated three times for each sample.

## 3. Results and Discussion

### 3.1. Thermal Stability

It is important to determine the thermal and oxidative stabilities of recycled materials, because the raw materials (recycled thermoplastic and rubber) were degraded during their service life, as well as during the recycling (grinding) processes. Some molecules are also prone to absorb moisture, which can influence the thermal stability of the resulting compounds [17]. It was observed that MAPE addition induced a positive effect on the blend compatibility by shifting the thermal decomposition of the composites to higher temperature as reported in previous works [17,20] (See Supplementary Materials: Table S1 and Figure S1).

### 3.2. Morphological Characterization

**Figure 1:**
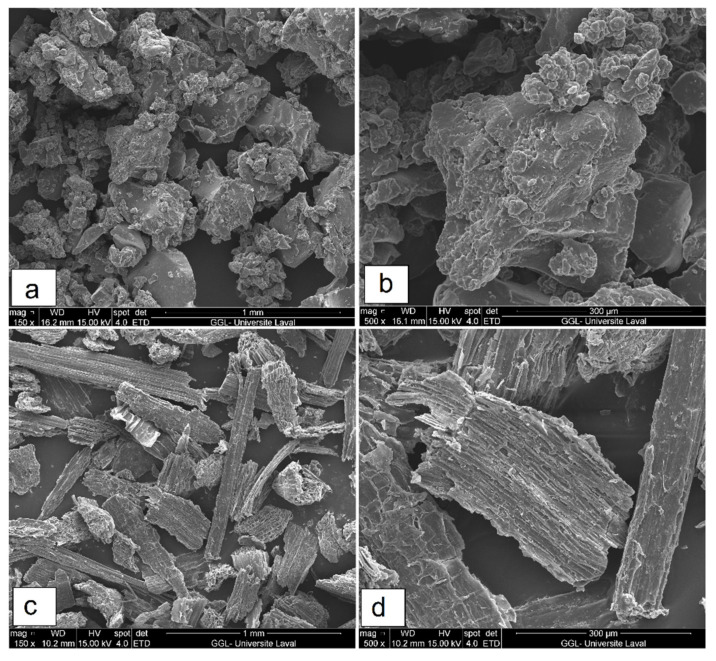
SEM micrographs of (**a**,**b**) RR and (**c**,**d**) FF particles at different magnification.

Figure 1 presents SEM micrographs of the flax fibers and recycled rubber particles at different magnifications. Recycled rubber particles show irregular shapes with protuberances or smooth angular surfaces caused by different grinding steps (downsizing) and different types of tires. Waste tire powders are composed of different recycled materials making it difficult to obtain a specific size and distribution. As shown in Figure 1c,d, the surface of FF is rough with sharp edges and covered by some fiber constituents (such as lignins and waxes) which may cause poor fiber wettability and adhesion by the matrix [5].

**Figure 2:**
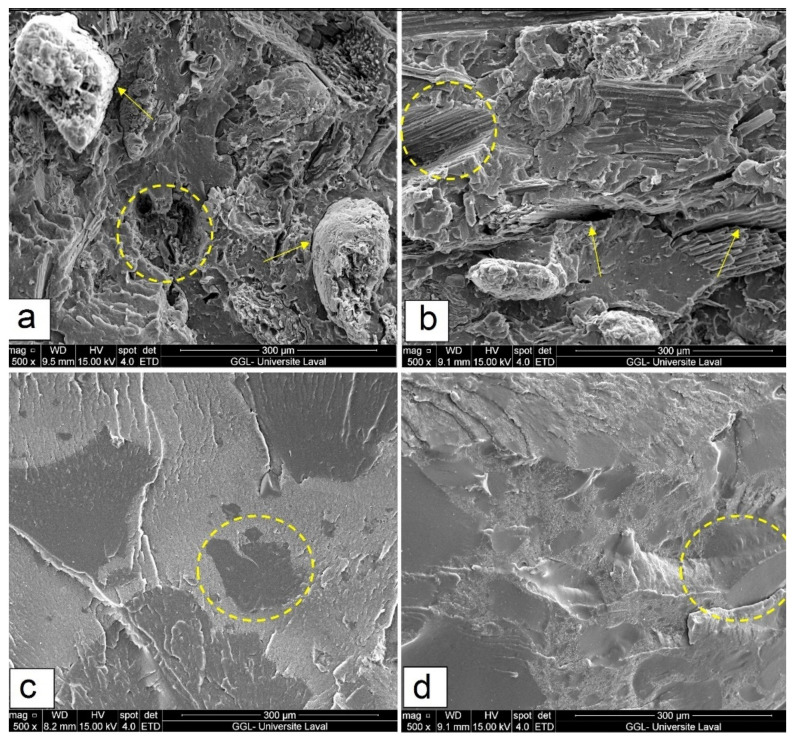
SEM micrographs of (**a**) F20, (**b**) F40, (**c**) R20 and (**d**) R40 composites (IM) (see Table 1 for definition).

Figure 2 presents the fractured surface of rHDPE/FF and rHDPE/RR blends (IM) for different filler ratios to compare the level of compatibility and interfacial adhesion between the particles and the matrix. Figure 2a shows poorly dispersed natural fibers within rHDPE, leading to easy pull-out due to improper stress transfer along the weak interface (typical behavior in NFC) leading to lower mechanical properties (strength), as described later. It is well documented that poor fiber wettability, due to the presence of lignin, hemicellulose, waxes and other fiber constituents, can limit interfacial adhesion in composites [5]. Several studies have reported similar findings for immiscible blends of thermoplastics filled with rigid particles [21,30]. On the other hand, poorly embedded RR particles in rHDPE showed clean and smooth fractured cross-section surfaces of binary rHDPE/RR blends attributed to incompatibility between the crosslinked rubber and rHDPE having low affinity and high interfacial tension resulting in easy pull-out of the dispersed particles (rubber phase) under stress (Figure 2d). Increasing the filler content caused filler agglomeration of weakly dispersed clusters and heterogenous morphology for F40 (Figure 2b) and R40 (Figure 2d) composites with void/crack formations at the interface region associated with high interfacial tension and incompatibility (phase separation), generating a higher number of defects (interfacial voids) and leading to fiber/rubber pull-out from the matrix [18,22,31].

**Figure 3:**
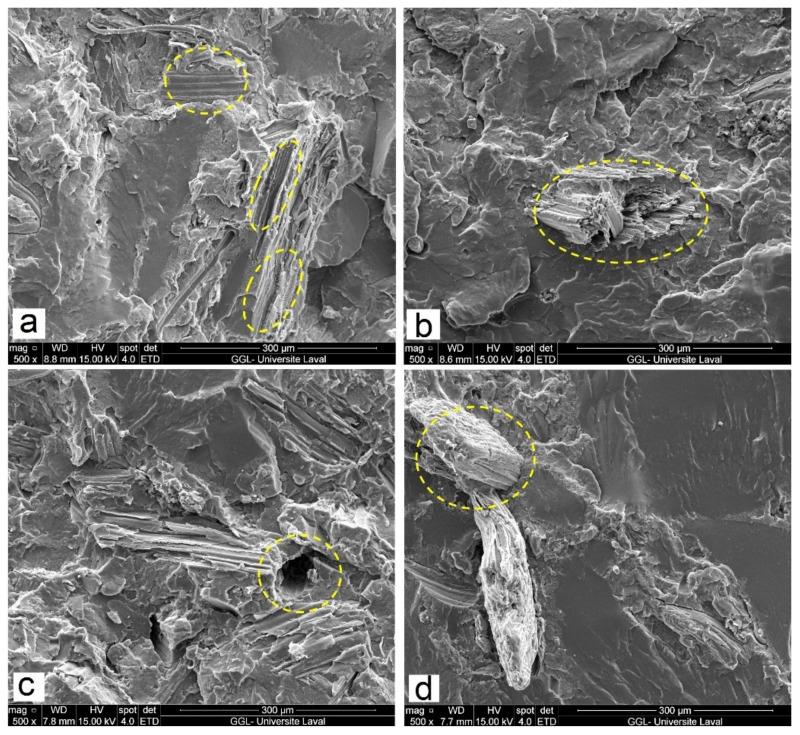
SEM micrographs of (**a**) R25F, (**b**) R25F*, (**c**) R40F and (**d**) R40F* (see Table 1 for definition).

SEM micrographs of the fractured surface for FF/RR-filled composites (IM) and compatibilized composites (IM) at 40 and 60 wt.% reinforcement for different FF/RR ratios (15/25 and 20/40) are presented in Figure 3. Again, low affinity between FF and RR with rHDPE caused catastrophic failure for uncompatibilized samples due to weak interfacial adhesion (Figure 2 and Figure 3), while the main reduction of blends homogeneity and failure strength occurred by increasing the filler content from 40 wt.% (Figure 3a) to 60 wt.% (Figure 3c). Phase morphology of multicomponent blends is determined by interfacial interactions and compatibility between the phases which are known to control the compound properties [18]. It is clear that adding MAPE improved the interfacial adhesion with the matrix (better compatibility) leading to a more homogeneous structure as very limited defects (holes, voids, cavities, agglomeration, etc.) can be seen. Figure 3b,d, show that 10 wt.% MAPE produced samples where the FF are still bonded to the matrix, while fiber pull-out almost completely disappeared due to the covalent links formed between the carbonyl groups of MAPE and the hydroxyl groups of the fibers, combined with chain entanglement of the nonpolar part of the compatibilizer (PE) with the matrix (rHDPE) [5]. Furthermore, interfacial gaps between fibers/rubber particles and the matrix are limited due to better distribution of RR coated by MAPE indicating improved compatibility as a result of chemical bonds formed between the maleic anhydride group of MAPE and the unsaturated C=C bonds on the rubber particles [32].

**Figure 4:**
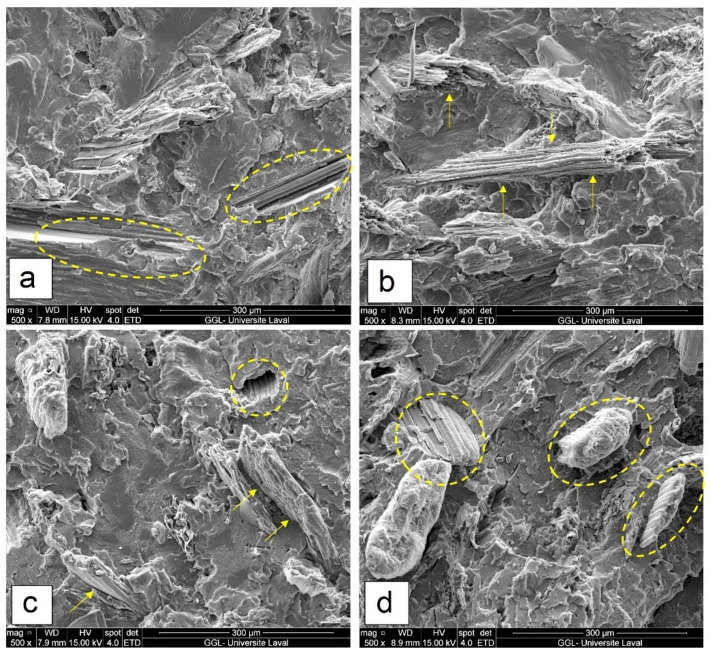
SEM micrographs of: (**a**) R40F* (CM), (**b**) R40F* (IM), (**c**) R55F* (CM) and (**d**) R55F* (IM) (see Table 1 for definition).

Figure 4 shows micrographs of the compatibilized composites prepared by CM and IM to determine the effect of the molding process. As shown in Figure 4a,c, the compression-molded specimens present poor surface interaction between FF and rHDPE (easy debonding and fiber pull-out) in R40F* and R55F* resulting from the formation of voids/cracks around the fibers. As for the injection molded samples (Figure 4b,d), they show better embedded fillers in the matrix, suggesting better dispersion and interaction between the components. This difference in morphology is expected to improve the interfacial stress transfer leading to improved mechanical properties. Comparing the fractured surfaces of both series, no particle agglomeration with fewer void/gaps around fibers was detected. The rough fractured surface for injection molded specimens suggests the positive effect of proper blending via IM on the homogenous mixture structure and uniform distribution of fillers. The gaps between the agglomerated particles and rHDPE in compression molded composites are clearly observed in Figure 4a,c. Similar results reported impregnation problem in compression molded hybrid composites attributed to the difficulty of high viscosity resins in diffusing into fiber bundles and subsequently their agglomeration (cluster) in highly viscous matrices [27,33]. High filler/matrix interaction may also explain the higher mechanical properties of injection molded composites due to more effective stress transfer and better interfacial quality.

### 3.3. Tension and Flexion Properties

In this work, it was possible to evaluate the mechanical properties up to 40 wt.% FF or 80 wt.% RR alone, or up to 80 wt.% for FF/RR mixtures. The tensile properties of the blends prepared by injection and compression molding are analyzed in terms of tensile strength, elongation at break, tensile modulus and flexural modulus.

**Figure 5:**
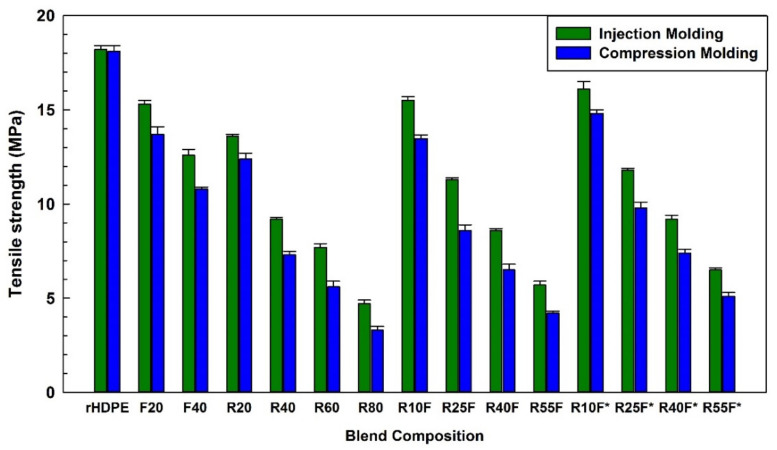
Tensile strength of the composites (see Table 1 for definition).

Figure 5 presents the tensile strength of binary blends of rHDPE/FF and rHDPE/RR, as well as rHDPE/(FF/RR) composites with different amounts of fillers with or without MAPE prepared by IM and CM. The tensile strength of IM samples dropped from 18.2 MPa (rHDPE) to 12.6 MPa with 40 wt.% FF revealing poor interaction between the inherently polar and hydrophilic FF with the non-polar and hydrophobic thermoplastic matrix causing poor interfacial stress transfer [5]. Moreover, the addition of 40 wt.% RR into rHDPE with limited entanglement between the matrix molecules and vulcanized rubber particles (stress concentration points) having low interface quality caused sharp (48%) tensile strength reduction for IM composites (18.2 to 9.2 MPa) [28]. When poor compatibility between the matrix and reinforcement occurs, the fillers (fiber and/or rubber) are easily pulled-out due to discontinuity (SEM images) and improper stress transfer from the matrix to the particles leading to low tensile strength [17]. As observed via SEM (Figure 5), the presence of MAPE improved the compatibility and filler dispersion leading to better stress transfer from the matrix to the particles; thus, higher tensile properties are observed for the compatibilized systems compared to uncompatibilized ones [20]. The maximum tensile strength was observed as of 16.1 MPa for R10F* (IM) because MAPE molecules were able to create interfacial bonds with both the FF and RR via reaction of its maleic anhydride groups with unsaturated C=C bonds of RR and hydroxyls groups (OH) available on the surface of FF, respectively. Moreover, the thermoplastic resin is a hydrophobic polymer, while FF are hydrophilic. So MAPE helps to reduce the polarity/hydrophilicity difference between the matrix and natural fibers leading to improved phase compatibility (more similar polarity and chemical bonding between the active groups) [34]. Similar results have been reported for fiber/rubber dispersion, and filler/matrix adhesion (interactions) using MAPE as a compatibilizer in rHDPE/maple wood fibers [35] and maleated polyethylene/GTR/wood flour composites [36]. For the processing method effect, the tensile strength of injection molded R40F* (9.2 MPa) is 20% higher than that of the compression molded sample (7.4 MPa) with the same composition. This can be attributed to the orientation of polymer chains during IM. In fact, direct injection of the specimens into the dumbbell-shaped mold induced chain orientation along the flow direction resulting in improved tensile properties along the longitudinal direction (anisotropy) [33]. Moreover, better distribution and dispersion of the fiber occurs due to a second melt-mixing step inside the injection molding screw barrel [37]. Similarly, Chuayjuljit et al. [38] observed higher tensile strength of compatibilized injected molded thermoplastic polyurethane (TPU)/wollastonite (100/40 phr) composites (15.8 MPa) compared to that of compression molded ones (9.4 MPa). This difference was attributed to a more uniform distribution of the 5 phr polypropylene grafted maleic anhydride (PP-g-MA) coupling agent in the matrix via IM resulting in better interfacial stress transfer.

**Figure 6:**
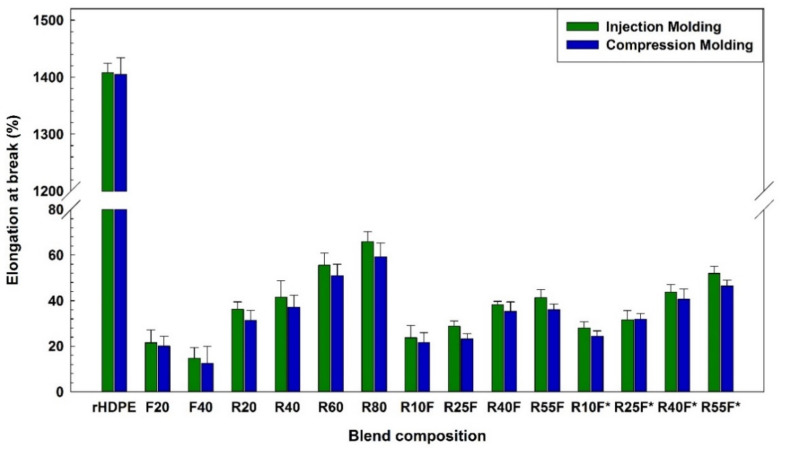
Elongation at break of the composites (see Table 1 for definition).

Flax fibers are relatively brittle short fibers with low elongation at break restricting the polymer chains of the thermoplastic matrix to slide one over the other resulting in low elongation at break of NFC [39]. As shown in Figure 6, low elongation at break of injection molded F20 (21.5%) and R20 (36.2%) compared to that of rHDPE (1408%) can be related to incompatibility of NFC and TPE systems leading to interfacial failure and poor load transfer between the phases [40]. It is well documented that addition of MAPE into incompatible blends presents a clear reduction in voids/defects and improving matrix/reinforcement interactions in terms of compatibility, adhesion and dispersion [41]. As can be seen from Figure 6, adding 10 wt.% MAPE into R55F increased the elongation at break of R55F* by 25% (41.3 to 51.9%) and 28% (36.1 to 46.4%) for samples prepared via IM and CM, respectively. It is claimed that MAPE improved compatibility through the interaction between the MA group of maleated copolymers as a polar component with the natural rubber (NR) (the main component of RR) as a nonpolar material [18], as well as chemical reaction between the active functional groups of the compatibilizer and the hydroxyl groups of FF [42].

**Figure 7:**
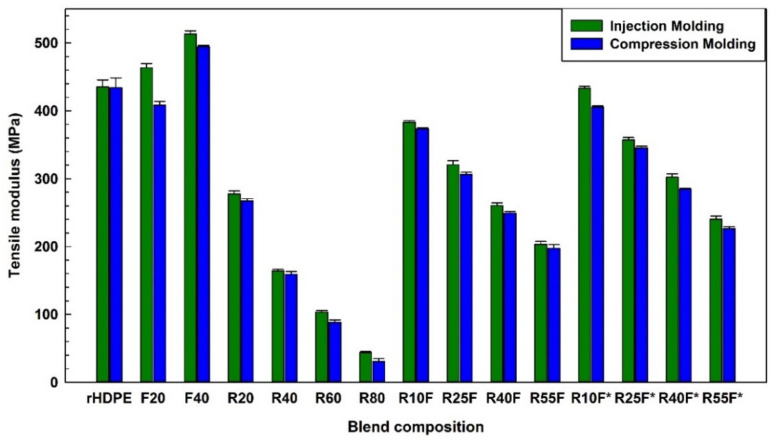
Tensile modulus of the composites (see Table 1 for definition).

**Figure 8:**
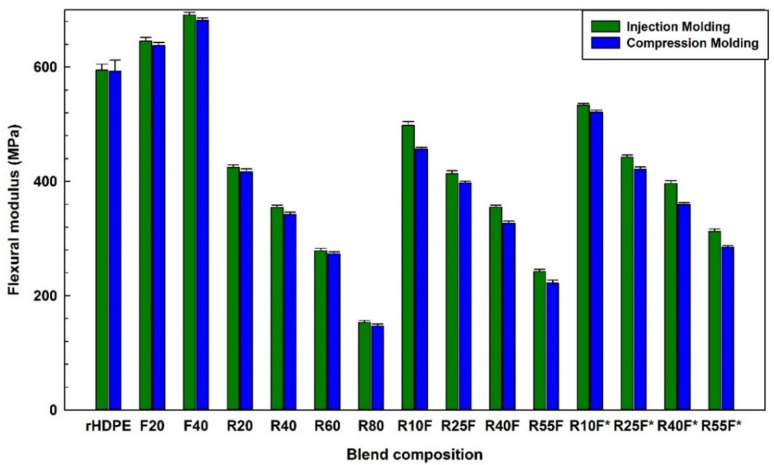
Flexural modulus of the composites (see Table 1 for definition).

As shown in Figure 7 and Figure 8, addition of FF (40 wt.%) increased the tensile and flexural moduli of injection molded composites by 17% (from 435.1 to 513.3 MPa) and 15% (from 594.5 to 691.1 MPa), respectively. These improvements can be related to the rigidity of natural fibers with high modulus (70 GPa) imparting higher stiffness and resistance to deformation [31]. Regardless of the molding method, addition of a soft rubber phase (low glass transition temperature) gives rise to a less rigid material with more elastic characteristics [18]. It must be pointed out that MAPE may promote surface crystallization to form a trans-crystalline layer around natural fibers with high rigidity and low deformability contributing to much higher modulus (tension/flexion) of compatibilized composites [43]. The higher tensile modulus of injection molded R25F (320.6 MPa) and R40F (260.2 MPa) samples than for compression molded R25F (306.4 MPa) and R40F (249.3 MPa) is again associated to the IM effect generating a more uniform reinforcement dispersion and better filler–matrix interaction under the applied stresses in agreement with similar reports [24].

### 3.4. Impact Strength

**Figure 9:**
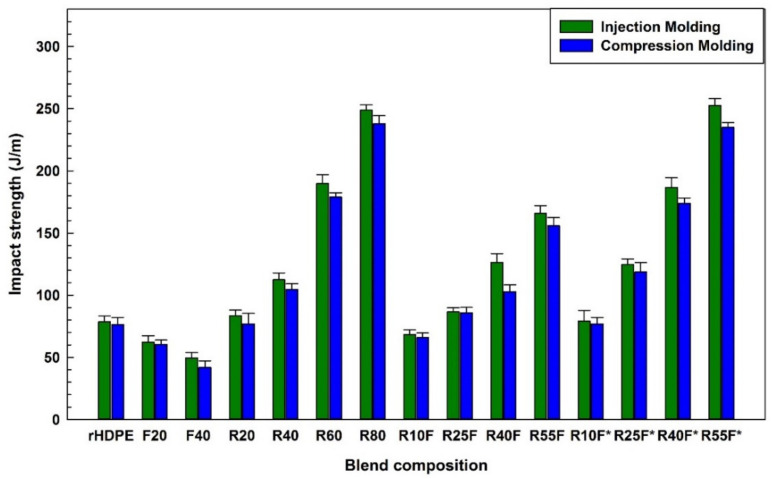
Impact strength of the composites (see Table 1 for definition).

As expected, the addition of natural fibers, such as FF, into thermoplastic matrices has a detrimental effect on the impact strength of NFC reducing the energy absorption capacity during fracture [14,20]. Figure 9 shows that the impact strength of NFC composites (IM) decreases with increasing FF content: from 62.3 to 49.6 J/m by adding 20–40 wt.% FF, due to structural defects/voids around rigid fibers and easy crack propagation along the weak interface. To solve this problem and improve the impact strength of NFC, waste tire rubber is used not only for impact modification purpose, but also to reduce environmental concerns (sustainability) and cost of the resulting parts by using less virgin raw materials. As shown in Figure 9, adding 40 wt.% RR particles into rHDPE improved the impact strength of R40 (IM) by 43% (78.6 to 112.4 J/m) which is associated with the presence of a soft rubber phase with partially crosslinked structure making the particles more deformable to absorb more energy and delay failure. However, the improved impact resistance upon increasing recycled rubber is at a cost of lower strength and modulus [14]. Figure 9 also shows that MAPE (10 wt.%) was able to compatibilize the rHDPE, FF and RR system, leading to a 34% impact strength increase (165.7 to 252.6 J/m) for injection molded R55F*. The presence of MAPE (compatible with the polyolefin matrix) surrounding FF and RR limited improve their dispersion (limiting particle-particle contact) resulting in a more uniform distribution of the applied stress thereby higher level of energy is required for fracture [20,44]. As shown in Figure 9 IM R40F* and R55F* have impact strengths of 186.8 and 252.6 J/m, respectively, which are, respectively, 7% and 9% higher than CM for the same composition. This difference is again related to better components mixing through a second melt blending step inside the IM screw, leading to better homogeneity and improved stress transfer between the phases. As observed in SEM images (Figure 2, Figure 3 and Figure 4), rubber particles agglomeration and interfacial voids can act as stress concentration points in CM samples, making crack initiation and propagation easier along these weak interfaces [27].

### 3.5. Hardness and Density

**Figure 10:**
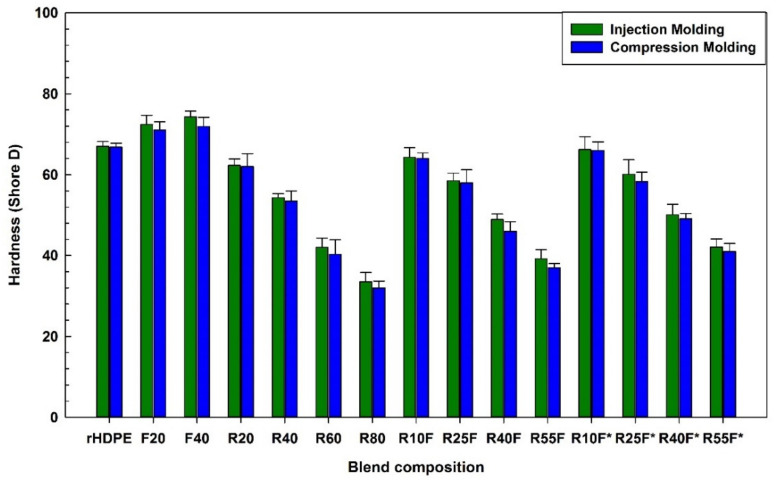
Hardness of the composites (see Table 1 for definition).

Figure 10 shows that the presence of FF and RR resulted in opposite trend of hardness values. For IM specimens, adding 40 wt.% FF as rigid particles increased the hardness from the matrix (67 Shore D) to 74 Shore D, while adding 40 wt.% RR decreased the hardness from 67 to 54 Shore D due to the soft nature of RR [28]. Furthermore, the rubber regeneration process is partially breaking down the crosslinked network of vulcanized rubber inducing even more flexibility (less rigidity) making RR filled blends softer due to improved chain mobility and lower crosslink density. There is also an effect of a processing oil used for rubber regeneration [28]. On the other hand, the introduction of FF (rigid fillers) in NFC increased the composites hardness in a similar way as tensile and flexural moduli (Figure 7 and Figure 8). Figure 10 shows that the hardness of the composites filled with a total filler content of 40 wt.% are in the order of: F40 > R25F* > R25F > R40. For the same composition, the specimens processed via IM and CM have similar hardness within experimental uncertainty.

The density of the raw materials can be classified in the order of: FF > RR > rHDPE > MAPE. Therefore, from density measurements, it was observed that adding FF (1.296 g/cm^3^) and RR (1.184 g/cm^3^) increased the density of composites due to their higher respective density compared to rHDPE (0.986 g/cm^3^). Moreover, the density of IM samples containing 40 wt.% of FF, RR or FF/RR are 1.086, 1.045 and 1.078 g/cm^3^, respectively, compared to rHDPE (See Supplementary Materials: Figure S2). Once again, no significant difference was observed between IM and CM in terms of density.

## 4. Conclusions

Despite significant potential of recycled tire rubber to modify the structure and properties of NFC, the number of studies on the production and characterization of fully recycled TPE reinforced by natural fibers is very limited. This study is devoted to the development of sustainable polymer composites using recycled plastics and tire residues reinforced with agro-waste. In particular, the effect of flax fiber (FF) and recycled rubber (RR) on the thermal stability, phase morphology and physico-mechanical properties (tensile, flexion, impact, hardness and density) of hybrid composites were investigated using recycled high-density polyethylene (rHDPE) as the matrix for total concentrations of up to 80 wt.%.

The effect of flax fiber (FF) and recycled rubber (RR) on the thermal stability, phase morphology and physico-mechanical properties (tensile, flexion, impact, hardness and density) of hybrid composites were investigated using recycled high-density polyethylene (rHDPE) as the matrix for total concentrations of up to 80 wt.%.

According to SEM results, binary rHDPE-based composites containing FF or RR showed low particle-matrix compatibility and poor filler distribution as filler aggregation was observed, especially with increasing FF content up to 40 wt.% and RR content up to 80 wt.%. However, the lack of affinity between both FF and RR with rHDPE was improved by adding MAPE (10 wt.%) as an adequate compatibilizing agent promoting interfacial adhesion and homogeneity.

Increasing the fiber content from 20 to 40 wt.% slightly increased the tensile (10%) and flexural (7%) moduli of rHDPE/FF, while the elongation at break (33%) and impact strength (20%) substantially decreased. On the other hand, increasing the RR particles (from 20 to 80 wt.%) increased the elongation at break (from 36.2 to 65.9%) which was attributed to the presence of an elastic phase inducing higher deformability/elasticity. Such increase was at the cost of lower stiffness and strength of the composites showing an inhomogeneous structure with poor RR dispersion in the polymer matrix with voids/cracks around the fillers facilitating fracture. However, the introduction of MAPE led to better dispersion and compatibility by forming an interfacial layer preventing particle-particle contact leading to less stress concentration points and significantly improving the tensile, impact and thermal properties. The injection molded R55F* showed the maximum elongation at break (51.9%) and impact strength (252.6 J/m) with improved thermal stability (higher decomposition temperature and higher char residues.

Finally, using injection molding (IM) resulted in better particle–matrix interaction (more homogenous morphology with a uniform distribution of well embedded reinforcements in the matrix) leading to higher elongation at break (51.9% for R55F*) and impact strength (252.6 J/m for R55F*) compared to compression molded (CM) samples (46.4% and 235 J/m for R55F*).

Overall, it can be concluded that natural fiber reinforced rubberized composites with good properties can be produced having the advantages of low cost and highly available raw materials. These compounds are believed to have potential industrial applications, such as automotive parts (bumper fascia, wiper blades and stone deflectors), and construction industries (beams, signboards and guardrails). There is also the possibility to reduce the costs via optimized process conditions (temperature, pressure, residence time and mixing steps). A feasibility study for industrial implementation of these materials is of high interest, including more advanced economics/life cycle/mechanical analyses.

## Figures and Tables

**Table 1:** Compositions investigated with their code.

Sample	rHDPE (wt.%)	FF (wt.%)	RR (wt.%)	MAPE (wt.%)
rHDPE	100	-	-	-
F20	80	20	-	-
F40	60	40	-	-
R20	80	-	20	-
R40	60	-	40	-
R60	40	-	60	-
R80	20	-	80	-
R10F	80	10	10	-
R25F	60	15	25	-
R40F	40	20	40	-
R55F	20	25	55	-
R10F*	80	10	10	10
R25F*	60	15	25	10
R40F*	40	20	40	10
R55F*	20	25	55	10

## Data Availability

Not applicable.

## Supplementary Materials

The following supporting information can be downloaded at: https://www.mdpi.com/article/10.3390/polym14153197/s1, Table S1: Decomposition temperatures (T10 and T50) and residues of the samples produced; Figure S1: Weight and derivative curves as a function of temperature for rHDPE, FF, RR, F40, R40, R25F and R25F* in: (**A**,**C**) air and (**B**,**D**) nitrogen; Figure S2: Density of the composites. References [5,17,20,21,45,46,47,48,49,50,51,52,53,54,55] are cited in the supplementary materials.
