# Supplementary material for: Recycled HDPE/Natural Fiber Composites Modified with Waste Tire Rubber: A Comparison between Injection and Compression Molding
Source: Polymers (Basel). 2022 Aug 5;14(15):3197. doi: 10.3390/polym14153197 (PMC9370949; doi:10.3390/polym14153197)
Supplement: Supplementary file 1 [file polymers-14-03197-s001.zip › polymers-1821274-supplementary.pdf]

Supporting Information

Recycled High-Density Polyethylene (rHDPE)/Natural Fiber Composites Modified with Waste Tire Rubber: A Comparison Between Injection and Compression Molding

1. Thermal stability

Figure S1 presents the TGA and DTG curves of the samples produced, while Table S1 reports the results in terms of residues, as well as T_{10} and T_{50}, which are the temperatures associated with 10% and 50% of weight loss, respectively. The initial weight loss of FF below 100 °C is ascribed to absorbed moisture, while dehydration and degradation of cellulose, hemicelluloses and lignin usually occur between 200 and 500 °C [45]. The thermal decomposition of GTR begins around 200 °C, so the processing temperature for these blends should not exceed 200 °C to avoid negative effects on the final TPE properties. The RR thermal decomposition in the range of 200 to 350 °C is associated with evaporation or decomposition of volatiles (processing oils, additives and other low molar mass compounds) in the RR formulation [46], while a wide bump between 350 and 430 °C can be related to the decomposition of the natural and synthetic rubbers having different decomposition temperatures [47]. The breakup of the crosslink network during rubber regeneration can thermally destabilize the rubber and promote its degradation at lower temperatures compared to GTR [48]. The DTG curve of RR in nitrogen presents a wide area between 350 and 430 °C comprised of two peaks ascribed to the differences in the decomposition temperature of natural and synthetic rubber [49]. The rHDPE has almost no branches in its molecular structure (HDPE) resulting in good thermal stability with a single strong peak between 400 and 450 °C in air that is shifted to 450-500 °C in nitrogen indicating slightly lower thermal stability in air related to oxidation [50].

Table S1 presents the thermal stability of the raw materials from the highest to the lowest values: MAPE > rHDPE > RR > FF. It is clear that $T_{10\,(air)}$ for RR (279 °C) and FF (273 °C) are very low compared to that of rHDPE (390 °C) and MAPE (394 °C) which contributes to earlier thermal decomposition of the compounds upon the introduction of both natural fibers and/or recycled rubber particles [51,52]. For example, incorporation of 40 wt.% FF, RR and FF/RR (15/25) mixture shifted $T_{10\,(air)}$ of rHDPE from 390 °C to 338, 345 and 342 °C under air. By comparison of FF and RR results, it can be concluded that increasing the RR content slightly increased the thermal stability of the compounds since thermal decomposition for the same filler content started slightly later and more residues were generated for RR containing samples compared to rHDPE/FF ones. For example, R40 shows 10% and 50% of mass loss at 345 °C and 436 °C in air, while T_{10} and T_{50} of F40 occurred at 338 °C and 415 °C in air suggesting better thermal stability of recycled rubber particles than that of natural fibers. Also, it is reported that the presence of carbon black in RR may serve as a physical barrier adsorbing low molecular weight (MW) volatile products produced during thermal degradation improving the thermal stability with increasing rubber content [53]. The ultimate weight losses are less than 100% as R25F produced 3.1% and 14.4% residues in air and nitrogen, respectively. These residues are associated with the presence of lignin in FF with a slow thermal decomposition combined with RR additives such as carbon black, silica and vulcanization additives (zinc oxide, antioxidant, processing oil, stearic acid and sulfur) used in the tire formulation [17,45].

As shown in Figure S1, the DTG curves of rHDPE/(FF/RR) composites in air show two main peaks. The first one is a small peak around 340-380 °C associated with the thermal decomposition of cellulose and/or hemicellulose of the fibers and NR decomposition, respectively. On the other hand, the other small peaks at 430 °C can be associated with SBR decomposition in RR [54]. The second main peaks in the DTG curves (460-510 °C) correspond to the thermal cracking of hydrocarbon chains (rHDPE matrix) [54].

It can be seen that MAPE addition induced a positive effect on the blend compatibility by shifting the thermal decomposition to higher temperature as reported in previous works [17,20]. Compared to neat rHDPE and compounds without compatibilizer, the thermal decomposition of R25F* shows that 10% and 50% of mass loss occurred at higher temperatures (364 °C and 460 °C) in air compared to nitrogen (370 °C and 476 °C) suggesting improved thermal stability with compatibilizer addition. Such improvement can be

associated with the higher level of interfacial bonding and the presence of a thin layer of MAPE with higher thermal stability surrounding the reinforcements and acting as a barrier layer increasing the overall thermal stability [5]. The formation of char residues can also slow down or inhibit the out-diffusion of the volatile decomposition products as a result of reduced permeability through a barrier effect of char residues formed between the heat source and polymeric compounds resulting in a more tortuous path decreasing the ultimate weight loss [45,52]. For example, the residues of R25F* (compatibilized with 10 wt.% MAPE) are 7.3% (air) and 26.5% (nitrogen), which are higher than the residues of R25F (3.1% in air and 14.4% in nitrogen). The compatibilization effect on the TPE thermal stability was also reported by Formela et al. [21]. They observed an increase in char residues of LDPE/GTR (50/50) blends by 39% (from 18.3 to 25.5 wt.%) upon the addition of a compatibilizer (SBS) creating a soft interface around the GTR particles improving interfacial adhesion and compatibility. The difference between the ultimate weight loss in air and nitrogen is associated with an additional oxidation step of carbon black to CO_2 around 540 °C leading to lower amount of residues in air [55].

Table S1. Decomposition temperatures (T_{10} and T_{50}) and residues of the samples produced.

**Sample**	**T_{10} (°C)**		**T_{50} (°C)**		**Residues (wt.%)**	
	Air	N_2	Air	N_2	Air	N_2
rHDPE	390	449	423	475	1.1	1.7
MAPE	394	456	442	463	0.3	0.8
RR	279	315	447	415	7.7	33.7
FF	273	278	340	364	7.2	9.8
F20	344	352	421	466	2.3	5.3
F40	338	347	415	461	2.2	5.9
R20	351	358	439	475	3.0	15.5
R40	345	350	436	474	3.3	16.8
R60	341	347	432	471	4.2	17.3
R80	337	343	430	468	5.6	19.9
R10F	345	355	434	473	2.6	10.6
R25F	342	354	433	471	3.1	14.4
R40F	340	345	430	465	3.9	16.6
R55F	339	340	428	461	4.9	17.5
R10F*	366	374	462	478	5.1	20.1
R25F*	364	370	460	476	7.3	26.5
R40F*	359	368	457	465	7.3	27.2
R55F*	352	366	451	463	7.9	28

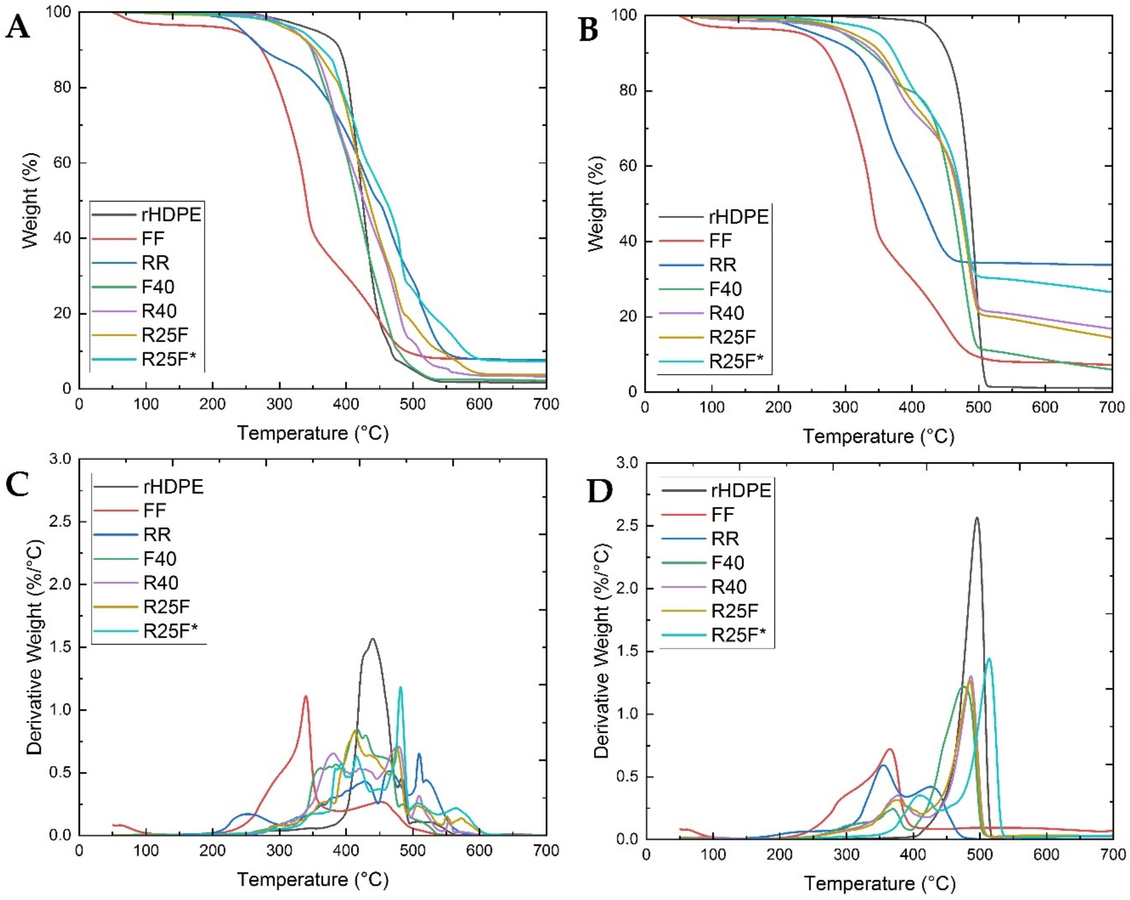

Figure S1. Weight and derivative curves as a function of temperature for rHDPE, FF, RR, F40, R40, R25F and R25F* in: (**A**,**C**) air and (**B**,**D**) nitrogen.

2. Density

Density as a function of FF and RR content for injection molded and compression molded composites in presented in Figure S2. As expected, the values increase with filler concentration. Within experimental uncertainty, there is no significant difference between both processing methods indicating that optimized conditions were used to produce all the samples.

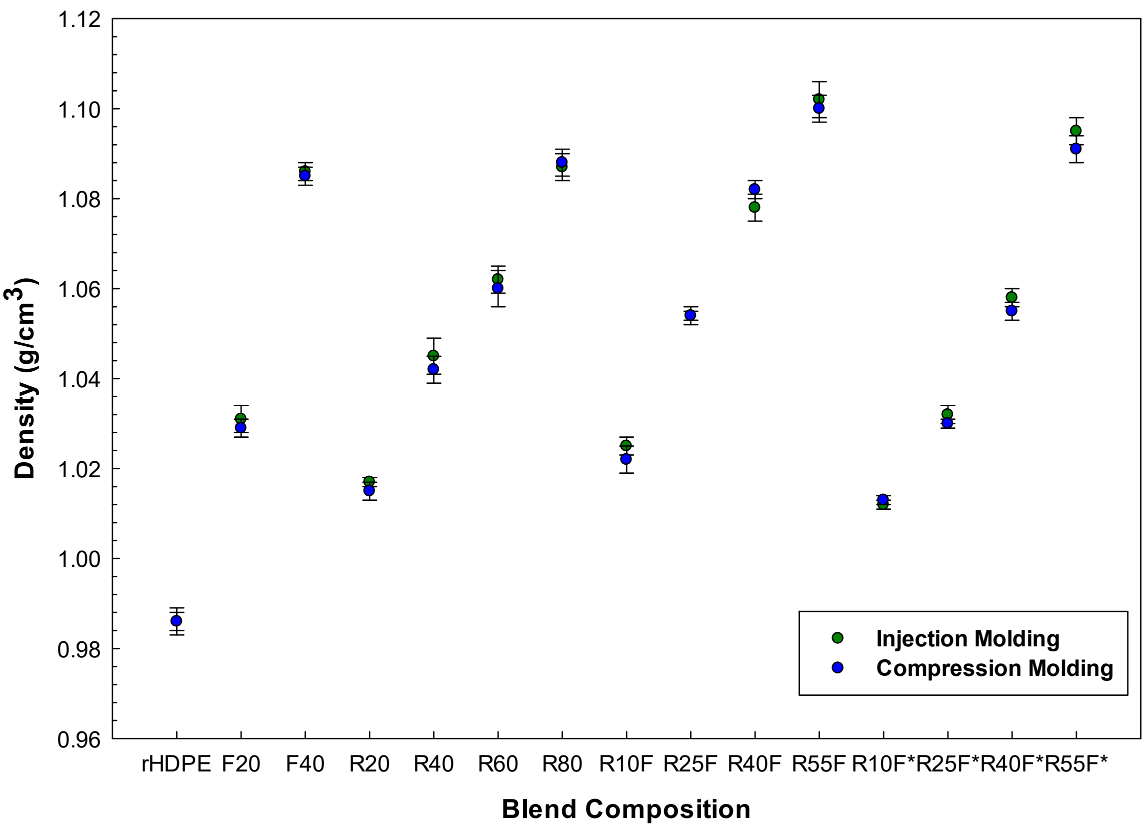

Figure S2. Density of the composites.
